# Spontaneous and evolutionary changes in the antibiotic resistance of *Burkholderia cenocepacia *observed by global gene expression analysis

**DOI:** 10.1186/1471-2164-12-373

**Published:** 2011-07-22

**Authors:** Andrea Sass, Angela Marchbank, Elizabeth Tullis, John J LiPuma, Eshwar Mahenthiralingam

**Affiliations:** 1 Organisms and Environment Division, Cardiff School of Biosciences, Cardiff University, Main Building, Park Place, Cardiff, Wales, CF10 3AT, UK; 2 University of Toronto, Department of Medicine, Division of Respirology, 1 King's College Circle, 6263 Medical Sciences Building, Toronto, ON, M5S 1A8, Canada; 3 University of Michigan Medical School, 1150 W. Medical Centre Drive, 8323 MRSB III, Box 0646, Ann Arbor, Michigan, MI 48109-646, USA

## Abstract

**Background:**

*Burkholderia cenocepacia *is a member of the *Burkholderia cepacia *complex group of bacteria that cause infections in individuals with cystic fibrosis. *B. cenocepacia *isolate J2315 has been genome sequenced and is representative of a virulent, epidemic CF strain (ET12). Its genome encodes multiple antimicrobial resistance pathways and it is not known which of these is important for intrinsic or spontaneous resistance. To map these pathways, transcriptomic analysis was performed on: (i) strain J2315 exposed to sub-inhibitory concentrations of antibiotics and the antibiotic potentiator chlorpromazine, and (ii) on spontaneous mutants derived from J2315 and with increased resistance to the antibiotics amikacin, meropenem and trimethoprim-sulfamethoxazole. Two pan-resistant ET12 outbreak isolates recovered two decades after J2315 were also compared to identify naturally evolved gene expression changes.

**Results:**

Spontaneous resistance in *B. cenocepacia *involved more gene expression changes and different subsets of genes than those provoked by exposure to sub inhibitory concentrations of each antibiotic. The phenotype and altered gene expression in the resistant mutants was also stable irrespective of the presence of the priming antibiotic. Both known and novel genes involved in efflux, antibiotic degradation/modification, membrane function, regulation and unknown functions were mapped. A novel role for the phenylacetic acid (PA) degradation pathway genes was identified in relation to spontaneous resistance to meropenem and glucose was found to repress their expression. Subsequently, 20 mM glucose was found to produce greater that 2-fold reductions in the MIC of multiple antibiotics against *B. cenocepacia *J2315. Mutation of an RND multidrug efflux pump locus (BCAM0925-27) and squalene-hopene cyclase gene (BCAS0167), both upregulated after chlorpromazine exposure, confirmed their role in resistance. The recently isolated outbreak isolates had altered the expression of multiple genes which mirrored changes seen in the antibiotic resistant mutants, corroborating the strategy used to model resistance. Mutation of an ABC transporter gene (BCAS0081) upregulated in both outbreak strains, confirmed its role in *B. cenocepacia *resistance.

**Conclusions:**

Global mapping of the genetic pathways which mediate antibiotic resistance in *B. cenocepacia *has revealed that they are multifactorial, identified potential therapeutic targets and also demonstrated that putative catabolite repression of genes by glucose can improve antibiotic efficacy.

## Background

*Burkholderia cepacia *complex (Bcc) bacteria are antibiotic resistant opportunistic pathogens known for their ability to infect individuals with cystic fibrosis (CF). The complex currently consists of 17 formally named species, of which *B. multivorans *and *B. cenocepacia *most often cause infection in CF [1]. The *B. cenocepacia *ET12 strain is one of the most problematic Bcc strains infecting patients with CF. It was most likely recognised as early as 1984 in the pioneering studies of Isles et al. [2] that showed that "*P. cepacia*" could cause an invasive, frequently fatal infection that became known "cepacia syndrome." Subsequent studies demonstrated that ET12 strains could transmit between CF patients by social contact [3], had spread intercontinentally across North America and Europe, and could replace infection with other Bcc species (reviewed [4]). Isolates of the ET12 strain can be identified using a number of characteristics including presence of the cable pilus gene and the *Burkholderia cepacia *epidemic strain marker, a RAPD 02 genotype fingerprint, a *recA *subgroup III-A phylotype (reviewed [5]), and most recently as possessing the multilocus sequence type (MLST) ST-28 [6]. As a result of the devastating CF infections caused by the *B. cenocepacia *ET12 strain, it has become one of the most studied Bcc bacteria in terms of virulence [7] and antimicrobial resistance [8,9].

The *B. cenocepacia *ET12 isolate, J2315, was recovered from an infected CF patient in Edinburgh, UK, in 1989 [3] and has been subject to complete genome sequence analysis [6]. This genomic resource enabled a *B. cenocepacia *microarray to be designed that has greatly enhanced our molecular understanding of this CF pathogen [10,11]. *B. cenocepacia *encodes multiple pathways that it uses to resist killing by antimicrobial agents, including efflux pumps [12], lipopolysaccharide, beta-lactamases and a trimethoprim resistant dihydrofolate reductase enzyme [6]. A problematic feature of chronic Bcc infection in CF is the ability of the infecting strain to adapt to very high levels of antibiotic resistance. The emergence of pan resistant Bcc strains, which are either untreatable or require combinations of multiple antibiotics to suppress exacerbations of infection, is of great concern [8]. The molecular mechanisms behind the evolution of spontaneous antimicrobial resistance in *B. cenocepacia *are not known and how the multiple resistance pathways function on a global scale to allow *B. cenocepacia *to survive antibiotic therapy is poorly understood. Cationic drugs such as chlorpromazine and theophylline have been shown to reduce the minimal inhibition concentration (MIC) of certain antibiotics that are otherwise ineffective against Bcc bacteria [13] and a more complete understanding of how these non-antibiotic drugs act may also provide novel therapies.

**Figure 1:**
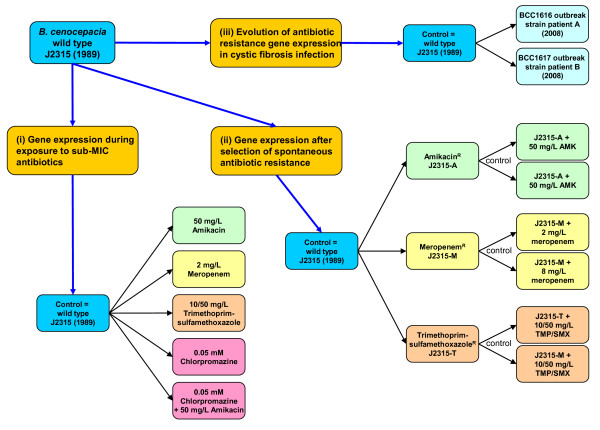
***B. cenocepacia *antimicrobial resistance traits examined by global gene expression**. The flow chart shows the three major questions addressed in regard to the antimicrobial resistance of *B. cenocepacia *(orange boxes). The microarray experiments performed to investigate these questions are shown with the control condition given in the blue box and test condition indicated as follows: amikacin (green boxes); meropenem (yellow boxes), trimethoprim-sulfamethoxazole (tan boxes); chlorpromazine (pink boxes); and the clonal outbreak strains (turquoise). The exception to the latter designation was that each spontaneously resistance mutant was used as the control condition when they were exposed to antibiotics.

In this study, we employed a transcriptomic, microarray-based strategy to investigate the following (Figure 1): (i) how exposure to sub-inhibitory concentrations of three antibiotics (amikacin, meropenem and trimethoprim-sulfamethoxazole) that are widely used to treat CF infections [14] and sub-inhibitory exposure to the cationic drug chlorpromazine affect gene expression in *B. cenocepacia*, (ii) how *B. cenocepacia *alters gene expression after the selection of spontaneous resistant mutants by the three latter antibiotics, and finally, (iii) how the antimicrobial resistance characteristics and global gene expression of the strain have naturally evolved during the 19 years it has circulated within the CF community since the isolation of the genome sequenced isolate, *B. cenocepacia *J2315 in 1989.

## Results and discussion

### Antibiotic susceptibility of B. cenocepacia J2315 and derived mutants with spontaneous resistance

Using the clinically defined MIC breakpoints, wild type *B. cenocepacia *J2315 was resistant to 15 of the 17 antibiotics tested; susceptibility to meropenem and ceftazidime was classified as intermediate (Table 1). Synergy testing indicated no synergy in any of the combination of two antibiotics tested against J2315. The antibiotic potentiator chlorpromazine reduced the MIC of amikacin (200 to 100 mg/L), tobramycin (150 to 50 mg/L) and azithromycin (50 to 15 mg/L) at a concentration of 0.2 mM; the same concentration of prochlorperazine produced slightly greater reductions in antibiotic MIC (amikacin to 20, tobramycin to 20 and azithromycin to 10 mg/L). These reductions were consistent with previous data obtained for "*B. cepacia*" strain ATCC 13945 [13] which is a *B. cenocepacia *IIIA strain, but not from the ET12 lineage (see http://pubmlst.org/bcc. As theophylline was not found to alter the susceptibility of strain J2315, chlorpromazine in combination with amikacin was selected as the optimal potentiator-antibiotic combination to examine from the *B. cenocepacia *global gene expression perspective because it had a good additive effect, did not present solubility problems in growth medium as seen with prochlorperazine, and allowed reproducible growth curves to be obtained for strain J2315.

**Table 1:** Antibiotic susceptibility (mg/L) of *B. cenocepacia *J2315, resistant mutants and outbreak isolates

Antibiotic clasBs	Antibiotic name	wild type J2315	J2315-A (AMK^r^)	J2315-M (MER^r^)	J2315-T (TMP^r^)	BCC 1616	BCC 1617
**Beta-lactams**	Meropenem	7	13	15	6	30	7

	Imipenem	125	125	175	100	175	100

	Ceftazidime	10	30	40	10	60	5

	Cefotaxime	250	500	1200	250	>1500	15

	Aztreonam	250	500	1500	200	>1500	40

	Piperacillin	450	450	900	350	1500	40

**Aminoglycosides**	Amikacin	200	450	200	200	450	900
	
	Gentamicin	700	5000	450	400	10000	>10000
	
	Tobramycin	150	500	300	125	1500	3000
	
							

**Macrolides**	Azithromycin	50	40	40	30	200	>500

	Erythromycin	150	150	200	150	500	800

**Folate synthesis inhibitors**	Trimethoprim/Sulfamethoxazole	30/150	20/100	30/150	150/750	10/50	20/100

**Fluoroquinolones**	Ciprofloxacin	7	7	8	30	>50	13

	Levofloxacin	7	7	8	30	>50	13

**Rifamycins**	Rifampicin	90	70	100	>200	60	80

**Chloramphenicol**	Chloramphenicol	10	10	25	>400	15	15

**Tetracyclines**	Tetracycline	60	70	180	120	160	>300

Spontaneous mutants with elevated resistance to amikacin (J2315-A), meropenem (J2315-M) and trimethoprim-sulfamethoxazole (J2315-T) were generated by plating on selective agar containing each respective antibiotic (see Methods). The mutants had MICs that were increased over the wild type, 2.3-, 2.1- and 5-fold, for each respective antibiotic (Table 1). The growth rate of each mutant was unaltered in comparison to J2315 and the resistant phenotype of each mutant was stable after 15 transfers on agar without antibiotics. The spontaneous mutants appeared at a frequency of 18.1 (± 7.9) × 10^-7 ^for amikacin, 4.5 (± 0.5) × 10^-7 ^for meropenem, and 14.4 (± 4.1) × 10^-8 ^for trimethoprim-sulfamethoxazole; no difference in mutation frequency was observed between J2315 cultures that were plated in log phase or at the beginning of stationary phase. Mutant J2315-A showed an increased resistance to all other aminoglycosides tested, and interestingly also to the beta-lactams meropenem, ceftazidime, cefotaxime and aztreonam (Table 1). The meropenem-resistant mutant, J2315-M, demonstrated elevated resistance to all beta-lactams tested, as well as increases in tobramycin, chloramphenicol and tetracycline resistance. Mutant J2315-T was more resistant to fluoroquinolones, chloramphenicol, rifampicin and tetracycline (Table 1).

### Antibiotic susceptibility and features of clonal outbreak strains circulating 19 years after isolation of B. cenocepacia J2315

Two isolates from two CF patients involved in a recent ET12 outbreak [15], BCC1616 and BCC1617, were compared to J2315 in terms of phenotype and global gene expression genotype. They represent the natural evolution of this epidemic strain as an infectious agent circulating in CF community since the recovery of J2315 in 1989. BCC1616 and BCC1617 were both found to be clonally identical with J2315 (MLST ST-28). Each was more resistant to aminoglycosides, macrolides, fluoroquinolones and tetracycline, than J2315 (Table 1); BCC1616 was less susceptible to all beta-lactams tested. This is in concordance with previous observations of rapid evolution of elevated drug resistance within sequential ET12 CF isolates, where isolates from episodes of clinical exacerbation were more resistant than those recovered during stable clinical conditions [9].

The growth rate of BCC1616 and BCC1617 on Iso-sensitest broth (doubling time = 3.0 and 2.4 hours, respectively) was also slower than J2315 (doubling time = 1.8 hours). *P. aeruginosa *isolates recovered from CF infection have reduced growth rates compared to those from other sources [16] and a slower growth rate is also linked to increased antibiotic resistance in bacterial pathogens [17]. Both of the recent ET12 clones were not motile in contrast to J2315 (Additional file 1, Figure S1). Loss of motility has been observed among *P. aeruginosa *isolates recovered from chronic CF infection [18]. Both J2315 and BCC1616 were auxotrophic for tyrosine and phenylalanine. Auxotrophy for amino acids has also been observed in CF isolates of *P. aeruginosa *and *B. cepacia *[19] and is attributed to the CF lung environment being rich in amino acids and other nutrients, selecting for auxotrophic variants. J2315 and BCC1616 both formed a brown pigment at the beginning of stationary phase on Iso-sensitest and on LB medium. *P. aeruginosa *CF isolates often produce a brown, melanin-like pigment, attributed to accumulation of homogentisate in the growth medium [16]. The same phenomenon has been observed for *B. cenocepacia *[20] with melanin production being correlated to increased resistance to oxidative stress. In contrast to J2315 and BCC1616, strain BCC1617 was not auxotrophic and did not produce a brown pigment, illustrating the known phenotypic variability of these bacteria [21].

### B. cenocepacia global gene expression in the presence of antibiotics

Transcriptomic analysis of strain J2315 was performed on mid-log phase cultures exposed to amikacin, meropenem, trimethoprim-sulfamethoxazole, chlorpromazine and a combination of amikacin and chlorpromazine (Table 2). Expression analysis of the spontaneous mutants was performed with and without each respective antibiotic on which the mutant had been selected; antibiotic exposure was performed at the same antibiotic concentration as used for J2315 and levels above this to induce further responses in the spontaneous mutants (Table 2). Validation of the microarray results was carried out by qPCR analysis of 17 protein coding genes of interest (Additional file 1, Table S1). In all cases the up- or downregulation observed by qPCR correlated to the microarray results corroborating previous studies using the *B. cenocepacia *J2315 microarray [10,11,22].

**Table 2:** Number of *B. cenocepacia *genes with a >2-fold change in expression

*B. cenocepacia *strain and growth condition	Number of genes significantly altered in expression (>2 fold):		
**Exposure of J2315 to sub-MIC antimicrobials:**	**Up-regulated**	**Down-regulated**	**Total**

J2315 wt + 50 mg/L AMK	17	2	19

J2315 wt + 2 mg/L MEM	53	6	59

J2315 wt + 10/50 mg/L TMP/SMX	39	17	56

J2315 wt + 0.05 mM CPZ	13	5	18

J2315 wt + 0.05 mM CPZ + 50 mg/L AMK	19	16	35

**J2315 resistant mutants:**			

J2315-A	26	28	54^a^

J2315-A + 50 mg/L AMK	32	50	82^a^

J2315-A + 250 mg/L AMK	37	38	75^a^

J2315-M	39	59	98^a^

J2315-M + 2 mg/L MEM	67	24	83^a^

J2315-M + 8 mg/L MEM	39	74	113^a^

J2315-T	41	39	80^a^

J2315-T + 10/50 mg/L TMP/SMX	44	38	82^a^

J2315-T + 60/300 mg/L TMP/SMX	52	53	105^a^

**ST28 outbreak isolates:**			

BCC1616	89	166	255

BCC1617	83	115	198

^a ^The number of genes differentially expressed were significantly different (p < 0.001; one-way analysis of variance with square root transformation) in the resistant mutants compared to the corresponding condition of J2315 exposed to each respective antibiotic.

Very few significant alterations in the expression of features on the microarray were observed after exposure of J2315 to amikacin (19 of 8740 probes; Table 2), while exposure to meropenem and trimethoprim-sulfamethoxazole resulted in approximately three times this number of features altering in expression (Table 2). The presence of 0.05 mM chlorpromazine also altered a limited number of features (18/8740), and this doubled with the combination of chlorpromazine and amikacin (Table 2). Overall, a greater number of genes were upregulated than downregulated in each antimicrobial exposure condition (Table 2). The number of differentially expressed genes was significantly higher in all experiments where the antibiotic resistant mutants were compared to J2315 (Table 2). The number of differentially expressed genes was highest in the outbreak isolates BCC1616 and BCC1617, with 255 and 198 differentially expressed genes compared to J2315 (Table 2).

### Gene expression in J2315 and J2315-A in response to amikacin

The upregulation of tRNAs dominated the transcriptomic response of J2315 to amikacin (15 of the 19 altering genes; Table 2); since this aminoglycoside targets protein synthesis, the alteration of tRNA expression may be correlated to the mode of action of amikacin but, further research will be need to validate this assumption. In terms of protein encoding genes only two were significantly upregulated in the presence of 50 μg/ml amikacin: BCAL1755, a protein with unknown function and the adjacent gene, BCAL1756, a CDS with homology to metal-dependent phosphohydrolases (Table 3). Both were also upregulated in J2315-A (Table 3). The induction of BCAL1756 by the presence of amikacin was confirmed by qPCR (Additional file 1 Table S1). BCAL1756 contains nucleotidyltransferase and phosphotransferase motifs that could possibly confer similar enzyme activities as those present in aminoglycoside-modifying enzymes, and which have been implicated in aminoglycoside resistance [23]. Introduction of the BCAL1755/BCAL1756 region into *B. vietnamiensis *G4 (see Additional file 1 Table S2 for construct information) which lacks homologous genes in its genome did not result in transformants with an elevated resistance to amikacin. Mobilization of the same construct into *B. cenocepacia *K56-2 (Additional file 1 Table S2), a genetically amenable *B. cenocepacia *strain that is frequently used as a surrogate model for genetic manipulation due to difficulties associated with J2315 [7], did not increase the amikacin resistance of this strain by insertion of additional copies this locus. The function of these genes was also tested by creating an isogenic knockout mutant in *B. cenocepacia *strain, K56-2 (Additional file 1 Table S2). In correlation with the gene transfer experiment deletion of genes BCAL1755-6 in mutant K56-2ΔL1755-6 did not result in increased susceptibility to aminoglycosides or any other antimicrobial tested (Table 4).

**Table 3:** Differentially expressed *B. cenocepacia *genes selected from all conditions tested

BExperimental condition from which the gene was selected		Fold change observed under each microarray condition			
**Gene name**	**Annotation**				

**Exposure to amikacin**		**J2315 wt + 50 mg/L AMK**	**J2315-A**	**J2315-A + 50 mg/L AMK**	**J2315-A + 250 mg/L AMK**

BCAL1233	Putative heat shock Hsp20-related protein	-	**1.95**	**3.47**	**4.92**

BCAL1234	Putative heat shock protein	-	-	**3.00**	**4.43**

BCAL1755	Conserved hypothetical protein	**2.44**	-	**4.74**	**3.47**

BCAL1756	Putative metal dependent phosphohydrolase	**1.78**	-	**2.95**	**3.14**

BCAL1919	ClpB heat-shock protein	-	-	-	**3.20**

BCAL2442	Chaperone protein HtpG	-	-	-	**2.39**

BCAL3146	60 kDa chaperonin 1	-	-	-	**2.47**

BCAL3147	10 kDa chaperonin 1	-	-	-	**2.96**

BCAL3148	Polyketide cyclase/dehydrase and lipid transport Family	-	**2.14**	**3.27**	**1.95**

BCAL3149	Outer membrane lipoprotein carrier protein LolA	-	**2.86**	**4.22**	**2.31**

BCAL3150	Putative exported protein	-	**3.15**	**4.34**	-

BCAL3151	putative transmembrane anti-sigma factor	-	**2.76**	**4.43**	**2.31**

BCAL3152	putative RNA polymerase sigma factor, ECF subfamily	-	**2.94**	**4.77**	**3.40**

BCAL3153	Putative lipoprotein	-	**3.47**	**5.82**	**4.45**

BCAL3270	Putative DnaK chaperone protein	-	-	-	**2.76**

BCAM0829a	RimL, Acetyltransferases, including N-acetylases of ribosomal proteins	-	**2.51**	**2.46**	**2.14**

BCAM0831	ABC transporter ATP-binding membrane protein	-	**1.91**	**2.43**	**3.01**

BCAS0637	60 kDa chaperonin 3	-	-	-	**6.62**

BCAS0638	10 kDa chaperonin 3	-	-	-	**7.88**

**Exposure to meropenem**		**J2315 wt + 2 mg/L MER**	**J2315-M**	**J2315-M + 2 mg/L MER**	**J2315-M + 8 mg/L MER**

BCAL0212	Putative phenylacetic acid degra-dation NADH oxidoreductase PaaE	-	-	-	**3.32**

BCAL0213	Phenylacetic acid degradation protein PaaD	-	-	-	**6.61**

BCAL0214	Phenylacetic acid degradation protein PaaC	-	-	-	**3.14**

BCAL0215	Phenylacetic acid degradation protein PaaB	-	**8.01**	**6.48**	**3.81**

BCAL0216	Phenylacetic acid degradation protein PaaA	-	**14.83**	**10.03**	**6.79**

BCAL0404	Phenylacetate-coenzyme A ligase	-	-	-	**2.58**

BCAL0405	Phenylacetic acid degradation protein PaaI	-	-	-	**2.75**

BCAL0406	Probable enoyl-CoA hydratase PaaG	-	**6.28**	-	**3.63**

BCAL0407	Beta-ketoadipyl CoA thiolase	-	-	-	**4.48**

BCAL0408	Putative phenylacetic acid degradation oxidoreductase paaZ	-	**9.93**	**7.93**	**5.75**

BCAL0409	Putative phenylacetic acid degra-dation enoyl-CoA hydratase PaaF	-	**2.67**	-	-

BCAL1804	Major Facilitator Superfamily protein	-	**-3.45**	**-2.70**	**-1.85**

BCAL1805	Putative sugar kinase	-	**-4.76**	**-4.35**	**-2.22**

BCAL1806	Conserved hypothetical protein	-	**-14.29**	**-9.90**	**-5.26**

BCAM1356	Putative gluconate 2-dehydrogenase subunit 3	-	**-25.64**	**-29.50**	**-12.85**

BCAM1357	Gluconate 2-dehydrogenase flavoprotein subunit	-	**-7.14**	**-7.30**	**-5.52**

BCAM1358	Gluconate 2-dehydrogenase cytochrome c subunit	-	**-24.69**	**-25.38**	**-9.80**

BCAM1710	Putative enoyl-CoA hydratase/isomerase	-	**3.23**	-	**4.32**

BCAM1711	Phenylacetate-coenzyme A ligase paaK	-	**12.48**	**11.01**	**5.24**

BCAM2165	Putative beta-lactamase, class A	**53.35**	-	**60.30**	**60.62**

BCAS0128	ABC transporter ATP-binding protein	**-2.27**	**-2.04**	-	**-3.70**

BCAS0129	Putative binding-protein-dependent transport system component	**-2.56**	**-2.13**	-	**-4.98**

BCAS0130	Putative ABC transporter substrate-binding protein	**-2.94**	-	**-2.48**	**-6.25**

BCAS0156	Beta-lactamase, class C	**116.10**	-	**133.10**	**61.12**

**Exposure to trimethoprim sulfamethoxazole**		**J2315 wt + 10/50 mg/L TMP/SMX**	**J2315-T**	**J2315-T + 10/50 mg/L TMP/SMX**	**J2315-T + 60/300 mg/L TMP/SMX**

BCAL0145	Adenosylhomocysteinase	**1.77**	-	-	**2.26**

BCAL0147	5,10-methylenetetrahydro-folate reductase	**1.61**	-	-	**2.07**

BCAM0186	Lectin	**2.90**	-	-	-

BCAM1869	Conserved hypothetical protein	-	**-3.68**	**-4.13**	**-2.09**

BCAM1870	N-acylhomoserine lactone synthase CepI	-	**-15.48**	**-14.95**	**-3.51**

BCAM1871	Conserved hypothetical protein	**2.03**	**-14.88**	**-12.90**	**-3.73**

BCAM2549	Multidrug efflux system outer membrane protein	-	**138.20**	**111.80**	**35.80**

BCAM2550	Multidrug efflux system transporter protein	-	**108.80**	**103.60**	**30.79**

BCAM2551	Multidrug efflux system transport protein CeoA	-	**296.90**	**341.50**	**55.38**

BCAM2552	Putative hydrolase	-	**187.40**	**167.50**	**45.93**

BCAM2554	LysR family regulatory protein	-	**4.34**	**4.36**	**2.40**

BCAS0292	Conserved hypothetical protein	**3.41**	**-6.02**	**-4.03**	-

BCAS0293	Nematocidal protein AidA	**8.56**	**-6.02**	**-5.65**	**-2.67**

BCAS0409	ZmpA	**1.99**	-	-	-

**Exposure to chlorpromazine**		**J2315 wt + 0.05 mM CPZ**	**J2315 wt + 0.05 mM CPZ + 50 mg/L AMK**		

BCAL1233	Putative heat shock Hsp20-related protein	-	**2.98**		

BCAL1234	Putative heat shock protein	-	**2.40**		

BCAL1755	Conserved hypothetical protein	-	**2.07**		

BCAL1756	Conserved hypothetical protein	**2.04**	1.82		

BCAM0923	Putative lipoprotein	**11.00**	**11.33**		

BCAM0924	Two-component regulatory system, response regulator protein	**4.85**	**4.63**		

BCAM0925	Multidrug efflux system outer membrane protein	**12.80**	**16.51**		

BCAM0926	Multidrug efflux system transporter protein	**17.75**	**27.45**		

BCAM0927	Multidrug efflux system transport protein	**13.75**	**22.43**		

BCAM2186	Putative macrolide-specific efflux system transport protein	1.96	**2.04**		

BCAM2187	Putative ThiJ/PfpI family protein	1.60	**2.02**		

BCAS0167	Squalene-hopene cyclase	**4.36**	**5.35**		

BCAS0168	TetR family regulatory protein	**2.33**	**2.81**		

BCAS0638	10 kDa chaperonin 3		**4.18**		

**Table 4:** *B. cenocepacia *K56-6 deletion mutants and their phenotype

Mutant name	Gene(s) deleted	Gene annotation	Relevant experiment/microarray observation	Phenotype^a^
K56-2ΔL1755-6	BCAL1755 BCAL1756	Hypothetical protein Phosphohydrolase	Upregulated in the presence of amikacin, in J2315-A and in BCC1617	None observed

K56-2ΔM0831	BCAM0831	ABC transporter	Upregulated in the presence of amikacin	None observed

K56-2ΔS0293-2	BCAS0292 BCAS0293	Hypothetical protein Nematocidal protein AidA	Upregulated in the presence of TMP/SMX	None observed

K56-2ΔM0924	BCAM0924	Response regulator protein	Upregulated in the presence of chlorpromazine	None observed

K56-2ΔM0925-7	BCAM0925 BCAM0296 BCAM0297	Multidrug efflux system OMP Multidrug efflux system protein Multidrug efflux system protein	Upregulated in the presence of chlorpromazine	Increased susceptibility for chlorhexidine (14 μg/ml, wt 22 μg/ml) Increased susceptibility for azithromycin (100 μg/ml, wt 150 μg/ml) Decreased growth rate in the presence of 1.0 mM chlorpromazine (μ = 0.49, wt μ = 0.55)

K56-2ΔM2186-8	BCAM2186 BCAM2187 BCAM2188	Efflux system transport protein ABC-type efflux carrier protein Outer membrane efflux protein	Upregulated in the presence of chlorpromazine	None observed

K56-2ΔS0167	BCAS0167	Squalene-hopene cyclase	Upregulated in the presence of chlorpromazine	Increased susceptibility for chlorhexidine (13 μg/ml, wt 22 μg/ml) Decreased growth rate in the presence of 1.0 mM chlorpromazine (μ = 0.62, wt μ = 0.80)

K56-2ΔS0081	BCAS0081	ABC transporter	Upregulated in outbreak strains BCC1616 and BCC1617	Increased susceptibility for chlorhexidine (14 μg/ml, wt 22 μg/ml) Increased susceptibility for tetracycline (100 μg/ml, wt 150 μg/ml)

^a ^The following antibiotics and antimicrobials were tested on all mutants, except K56-2ΔS0167: amikacin, tobramycin, meropenem, ceftazidime, trimethoprim, azithromycin, erythromycin, levofloxacin, tetracycline, chloramphenicol, chlorhexidine

The amikacin-resistant mutant J2315-A demonstrated upregulation of different genes/gene clusters compared to the wild type, regardless of the present or absence of the antibiotic (Table 3). Most conspicuous was the upregulation of a gene cluster on the large chromosome, BCAL3148-3153. One of these genes, BCAL3152 encoded a sigma factor (extracytoplasmatic function subfamily; ECF) and an adjacent anti-sigma factor, BCAL3151. The BCAL3152 sigma factor is highly conserved among *Burkholderia *species [24]. ECF sigma factors are involved in sensing and regulating the response to changes in the environment, including maintaining cell envelope integrity under stress [25]. The BCAL3148-3153 cluster was not induced in the wild type by sub-inhibitory concentrations of amikacin, but its constitutive upregulation in J2315-A suggested it may play a major role in the general stress response linked to the spontaneous adaptation to an elevated state of antimicrobial resistance.

Another gene cluster found to be constitutively upregulated in J2315-A was a putative acetyltransferase, BCAM0829a, and a nearby ABC transporter gene, BCAM0831 (Table 3). Limited upregulation of BCAM0829a was observed when J2315 was exposed to amikacin (Table 3) but this was confirmed by qPCR (Additional file 1 Table S1). Acetyltransferases are known mediators of aminoglycoside resistance [23] and ABC transporters often contribute towards multi-drug resistance in *E. coli *[26], hence, both genes, BCAM0829a and BCAM0831 have plausible roles in functionally mediating resistance to amikacin. However, transfer of the acetyltransferase BCAM0829a into *B. vietnamiensis *G4 or *B. cenocepacia *K56-2 failed to alter their susceptibility to amikacin, gentamicin and tobramycin (with or without chloropromazine), and deletion of the associated ABC transporter gene BCAM0831 in mutant K56-2ΔM0831 also did not reveal an antimicrobial phenotype (Table 4).

### Gene expression in J2315 and J2315-M in response to meropenem

The global gene expression response of *B. cenocepacia *to meropenem was characterised by upregulation of predictable factors such as beta-lactamases and of novel enzymes like those linked to the phenylacetic acid (PA) degradation pathway (Table 3). BCAS0156, a class C beta-lactamase encoded on the small chromosome was the most upregulated gene seen when the wild type was exposed to meropenem; a class A beta-lactamase, BCAM2165, encoded on the medium chromosome was also highly expressed (Table 3). Interestingly, neither beta-lactamase was significantly altered in expression in the meropenem resistant mutant in the absence of the antibiotic (Table 3), indicating that these enzymes were not primarily involved in the elevated resistance phenotype of J2315-M (Table 1).

Located 135 bp upsteam of the class A beta-lactamase BCAM2165 was a LysR family regulator BCAM2166, with 90% homology to and in a location corresponding to that of *penR *previously described in "*P. cepacia" *[27] (now known to be a *B. multivorans *strain). This region is conserved in all completed *Burkholderia *genomes and Trépanier et al. [27] showed that *penR *controlled the transcription of the class A beta-lactamase *penA *with a putative binding site mapped to a region 130 bp upstream of the beta-lactamase, directly adjacent to the regulator sequence. A motif search revealed the same binding site in J2315, upstream of both BCAM2165 and BCAS0156, suggesting that they may be co-regulated and possibly under the control of the BCAM2166 regulator.

Although both beta-lactamases were upregulated in the presence of meropenem, it was not known which, if any, is active against the antibiotic. Carbapenem hydrolyzing class A beta-lactamases have been reported for *Klebsiella pneumoniae *[28]. A class A *penB *beta-lactamase has been characterised in *B. cenocepacia *[29] that is active against penicillin and cephalosporins, but has only a marginal ability to degrade meropenem and imipenem. A plasmid-encoded class C beta-lactamase with weak imipenemase activity has also been reported [30]. To clarify the involvement of BCAM2165 in meropenem inactivation by *B. cenocepacia*, clavulanic acid was tested as a class A-specific beta-lactamase-inhibitor, but it failed to reduce the MIC for meropenem even at concentrations as high as 64 mg/L (data not shown). The role of the class C beta-lactamase, BCAS0156 was tested by complementation into *B. vietnamiensis *G4 which lacks this gene (see Additional file 1 Table S2 for construct). The resulting transformant showed an increase in MIC for cefotaxime (from 8 to 35 mg/L), ceftazidime (from 7 to 50 mg/L), imipenem (from 8 to 40 mg/L) and piperacillin (from 4 to 15 mg/L), but the MIC for meropenem (0.35 mg/L) and aztreonam (6 mg/L) remained unchanged from that of wild type *B. vietnamiensis *G4. When the same construct was mobilized into *B. cenocepacia *K56-2, the MIC for cefotaxime (300 to 400 mg/L) and ceftzidime (40 to 45 mg/L) increased, but the remaining antibiotic MICs were unaltered. Addition of chlorpromazine to the complemented strains did not alter their antibiotic susceptibility further. A functional role for BCAS0156 in mediating high level beta-lactam resistance was therefore supported, but interestingly not for meropenem as a specific substrate for this class C beta-lactamase.

Three gene clusters annotated as phenylacetic acid degradation enzymes [31,32] were upregulated in mutant J2315-M regardless of the presence or absence of the meropenem (Table 3). In *B. cenocepacia*, these genes are organised as follows: two gene clusters on the large chromosome (BCAL0212-0216, BCAL0404-0409) and one gene cluster on the medium chromosome (BCAM1710-1712). All three clusters appeared largely co-regulated from the microarray data and a MEME search revealed a common regulatory motif [33] for all three locations, upstream of BCAL0216, BCAL0408 and BCAM1712 respectively. A MAST analysis revealed the same regulatory motif to be present upstream of the PA degradation genes in all genome sequenced *Burkholderia *species. Upregulation of BCAL0216, BCAL0408, and BCAL1711/12 was confirmed by qPCR (Additional file 1 Table S1).

Two sets of genes involved in glucose transport and metabolism via the pentose phosphate pathway were substantially down regulated in the resistant mutant J2315-M: BCAL1804-1806 and BCAM1356-1358 (Table 3). Growth curve analysis showed that J2315-M grew at less than half the growth rate on glucose than J2315. The PA catabolic enzymes have been shown to be under glucose catabolite repression in *B. cenocepacia *[33]. Our data therefore suggested that for J2315-M to achieve the upregulation of PA genes and increase its meropenem resistance, it had to down regulate the glucose transport and metabolism genes to lower the intracellular glucose concentration. The resulting slower growth rate of J2315-M in presence of glucose may also be an advantageous phenotype for surviving antimicrobial exposure, analogous to the alterations seen in small colony variants [34].

To elucidate if the PA catabolic enzymes aid resistance to antibiotics, MIC values in the presence and absence of glucose (20 mM) in LB medium were determined. The presence of glucose resulted in reductions of ≥ 2-fold in J2315's MIC for meropenem (50 to 15 mg/L), ceftazidime (275 to 160 mg/L), chloramphenicol (16 to 8 mg/L) and tetracycline (50 to 10 mg/L); no change was observed for amikacin, tobramycin, erythromycin, levofloxacin and trimethoprim-sulfamethoxazole. A panel of 17 closely related *B. cenocepacia *were also tested and 7 out of 17 also demonstrated a reduction in meropenem MIC in the presence of 20 mM glucose (data not shown). Analysis of PA gene expression directly correlated to these phenotypic observations, being considerably reduced in J2315 (48-fold by qPCR for BCAL0216) in the presence of glucose. Expression of this gene was unaltered by addition of glucose for *B. cenocepacia *K56-2, which did not demonstrate the same MIC reduction for meropenem.

The strain to strain variability of this putative catabolite repression response suggest that the regulation of central metabolism in *B. cenocepacia *is highly complex; given the number of paralogous metabolic pathways present in *Burkholderia *genomes [35] such plasticity is not surprising. qPCR showed that the expression of the beta-lactamases (BCAM2165 and BCAS0156) was not affected by the presence of glucose in J2315 indicating that these degradative antibiotic resistance enzymes were not catabolite repressed. The role of the PA degradation enzymes in *Burkholderia *is not yet fully understood, but recently it was shown that PA pathway is necessary for full pathogenicity during *Caenorhabditis elegans *infection [36]. The linkage of PA degradation to the phenomenon of spontaneous antibiotic resistance in *B. cenocepacia *adds further complexity to the central role aromatic metabolism plays in the biology of these microorganisms.

### Gene expression in J2315 wt and J2315-T in response to trimethoprim-sulfamethoxazole

The gene encoding a homologue of AidA (BCAS0293), a quorum sensing regulated gene required for full virulence of *B. cenocepacia *H111 during *C. elegans *infection [37] was the most upregulated gene when wild type J2315 was exposed to sub-inhibitory levels of TMP-SMX (Table 3). Other quorum sensing regulated genes such as the zinc metalloprotease [38], ZmpA (BCAS0409), and the lectin encoding BCAM0186 [11] were also upregulated. Few other significant alterations were made by J2315 in response of sub-lethal amounts of TMP-SMX (Table 3). Deletion of BCAS0293 together with the adjacent BCAS0292 in mutant K56-2ΔS0293-2 did not alter its susceptibility to trimethoprim, sulfamethoxazole, or any other antimicrobials (Table 4).

The TMP-SMX resistant mutant J2315-T also altered the expression of a limited number of genes, however, the most conspicuous change it made was a strong and constitutive upregulation of an efflux pump of the RND family, BCAM2549-2552 (Table 3). This is the J2315 homologue of the ceo multidrug efflux pump operon described by Nair et al. [39] consisting of the three parts of the efflux pump (BCAM2549-2551) and a hydrolase/lipase (BCAM2552). The MIC data for J2315-T showed increased resistance to trimethoprim, fluorquinolones, chloramphenicol and tetracyclines (Table 1; all are known substrates for this pump), a phenotype consistent with the substrate spectrum of the ceo efflux pump described by Nair et al. [39] and thus corroborating the microarray expression data. Upstream and divergently transcribed from the ceo efflux pump is a LysR family regulatory protein, BCAM2554, that is a homologue of the *ceoR *transcriptional activator [39]. This regulator was constitutively upregulated in mutant J2315-T (Table 3). Nair et al. [39] had also shown that the pump was upregulated in the presence of salicylate. In contrast to their findings made on *B. cenocepacia *strain PC121 [39], where salicylate and chloramphenicol were found to induce expression of the ceo efflux pump, we found that both the efflux pump and *ceoR *in *B. cenocepacia *J2315 and K56-2 were not inducible by salicylate (as tested by qPCR, data not shown) or sub-inhibitory levels of TMP-SMX (Table 3).

### Gene expression in J2315 wt in response to chlorpromazine

Exposure of *B. cenocepacia *to this potentiator of aminoglycoside activity resulted in significant upregulation of two efflux gene clusters, BCAM0923-0927 and to a lesser extent, BCAM2186-2188 (Table 3). No specific link to amikacin-related gene expression, the antibiotic most potentiated by chlorpromazine, was observed (Table 3). The BCAM2186-2188 pump had homology to a macrolide-specific ABC-type efflux pump system in *E. coli *[26]. Transfer of the efflux pump genes to *B. vietnamiensis *G4 or *B. cenocepacia *K56-2 (Additional file 1 Table S2) did not alter the antimicrobial susceptibility of these strains (either with or without the addition of chlorpromazine). Likewise creation of a *B. cenocepacia *deletion mutant K56-2ΔM2186-8 did not produce an observable phenotype in relation to the antibiotics (with or without chlorpromazine) examined in this study (Table 4).

In relation to the other chlorpromazine activated efflux system, BCAM0926 and BCAM0927, were closely related to MexD and MexC, respectively, components of the MexCD-OprJ efflux pump in *P. aeruginosa *[40]. BCAM0925, an outer membrane protein, is most similar to OprM, which is part of the MexAB-OprM efflux pump in *P. aeruginosa *[40]. BCAM0924 is the response regulator part of a two component regulatory system that may control expression of the efflux pump; its role was investigated by the construction of a deletion mutant K56-2ΔM0924 (Table 4). The mutation did not alter its chlorpromazine or antibiotic MIC (Table 4). The expression of the structural pump genes, BCAM0926-0927, was also still induced by chlorpromazine in the deletion mutant suggesting that BCAM0924 was not critical for regulation of the adjacent efflux pump (data not shown).

To further investigate the role of the efflux pump structural genes BCAM0925-0927, a K56-2 deletion mutant spanning all 3 genes was constructed (Table 4). The K56-2ΔM0925-7 mutant demonstrated increased susceptibility to the disinfectant chlorhexidine and the antibiotic azithromycin, but no alteration in susceptibility to other antibiotics. Growth curve analysis of the K56-2ΔM0925-7 mutant revealed a 10% slower growth rate in the presence of chlorpromazine compared to the parent strain. qPCR analysis of the K56-2 parent strain showed that chlorhexidine, as well as chlorpromazine induced the efflux pump gene BCAM0927, 345-fold in the presence of 5 μg/ml chlorhexidine and 125-fold in the presence of 0.05 mM chlorpromazine. Chlorhexidine, like chlorpromazine is a cationic compound known to interact with and damage cellular membranes [41], hence it is logical that both compounds induce the efflux pump and that the efflux mutant demonstrated sensitivity to this disinfectant. Chlorhexidine is also known to induce the *Pseudomonas aeruginosa *MexCD-OprJ efflux pump [41]. Since the BCAM0925-0927 efflux operon shares considerable homology with latter *P. aeruginosa *system, the mutant phenotype also suggests that the pump may play a role in chlorhexidine efflux in *B. cenocepacia*. Why deletion of the pump led to an alteration in susceptibility to azithromycin is not known.

Another gene cluster upregulated in the presence of chlorpromazine was a squalene-hopene cyclase, BCAS0167, and an adjacent, divergently transcribed response regulator, BCAS0168 (Table 3). Squalene-hopene cyclase is involved in the synthesis of hopanoids, the prokaryotic equivalent of steroids [42] which may function to counter the effect of membrane disrupting agents on bacterial cells. A deletion mutant of BCAS0167, K56-2ΔS0167, demonstrated reduced growth rate in the presence of chlorpromazine and chlorhexidine, as well as an increased susceptibility to chlorhexidine (Table 4). Since the squalene-hopene cyclase gene and surrounding region is highly conserved in several Bcc genomes (*B. cenocepacia*, *B. multivorans, B. lata *and *B. ambifaria*) these data suggest it plays an integral role in the intrinsic resistance of *Burkholderia *to membrane disrupting antimicrobial agents.

### Gene expression in the pan-resistant CF outbreak isolates BCC1616 and BCC1617

The most pronounced change in expression profiles of both ET12 outbreak isolates was the downregulation of genes involved in flagella production and in chemotaxis (Table 5) compared to J2315. BCC1616 and BCC1617 also downregulated expression of their cable pilus genes (Table 5). The lack of flagella and chemotaxis gene expression correlated directly to the non-motile phenotype of the outbreak isolates, in contrast to J2315 which was motile (Additional file 1, Figure S1). During rapid growth in a sputum-based medium J2315 upregulates genes involved in production of the flagellum [10]. This may reflect a short term response of *B. cenocepacia *to early growth during infection, where flagella and motility serve as important virulence factors [43]. Lack of motility has been observed in *P. aeruginosa *CF isolates from chronic infection [18] and as a global gene expression adaptation to growth in sputum [44]. The stable non-motile phenotype and genotype of the recent ET12 clinical strains suggest that *B. cenocepacia *may in time also adapt to the same phenotype associated with *P. aeruginosa *from chronic CF infection.

**Table 5:** Selected genes differentially expressed in the recent *B. cenocepacia *outbreak strains BCC1616 and BCC1617 compared to *B. cenocepacia *J2315

Functional class and gene name	Annotation	Fold change in expression^1)^	
		
		BCC1616	BCC1617
**Motility and chemotaxis**			

BCAL0113	B-type flagellar hook-associated protein 2	**-18.80**	**-11.33**

BCAL0114	flagellin (type II)	**-38.91**	**-24.51**

BCAL0126	chemotaxis protein MotA	**-3.22**	**-2.41**

BCAL0127	chemotaxis protein MotB	**-5.68**	-

BCAL0128	chemotaxis two-component response regulator CheY	**-6.76**	**-2.95**

BCAL0129	chemotaxis two-component sensor kinase CheA	**-6.80**	**-3.26**

BCAL0130	chemotaxis protein CheW	**-3.11**	**-2.82**

BCAL0521	flagellar FliJ protein	**-4.31**	**-3.28**

BCAL0576	flagellar hook-associated protein 1 (HAP1)	**-11.57**	**-5.38**

BCAL0577	flagellar hook-associated protein 3 (HAP3)	**-14.33**	**-6.21**

BCAL0762	putative methyl-accepting chemotaxis protein	**-3.32**	**-2.99**

BCAM1503	putative methyl-accepting chemotaxis protein	**-16.92**	**-9.90**

BCAL1662	putative methyl-accepting chemotaxis protein	**-4.98**	**-2.57**

BCAM1804	methyl-accepting chemotaxis protein	**-12.56**	**-6.71**

BCAM2564	putative aerotaxis receptor	**-6.45**	**-3.52**

**Cable pilus biosynthesis**			

BCAM2758	two-component regulatory system, sensor kinase protein	**-2.19**	**-2.63**

BCAM2759	putative minor pilin and initiator	-	**-3.69**

BCAM2760	putative outer membrane usher	**-2.15**	**-2.96**

BCAM2761	giant cable pilus	**-2.97**	**-6.90**

BCAM2762	giant cable pilus chaperone protein	**-4.44**	**-10.00**

**Transport, efflux and antimicrobial resistance**			

BCAL1674	multidrug efflux system AmrA protein	-	**2.49**

BCAL1756	Putative metal dependent phosphohydrolase	-	**1.97**

BCAM2165	putative beta-lactamase, class A	**2.45**	-

BCAS0081	ABC transporter ATP-binding membrane protein	**35.39**	**33.37**

BCAS0082	Hydrolase of the alpha/beta superfamily	**41.13**	**30.37**

**Restriction modification**			

BCAL0414_J_1	type I modification component of restriction-modification system (pseudogene)	**23.04**	**24.84**

BCAL0418	type I restriction enzyme specificity protein	**12.59**	**10.77**

BCAL0420	type I restriction component of type I restriction-modification system	**3.79**	**3.04**

**Transposition**			

BCAL0044	putative transposase	**3.75**	**2.91**

BCAM2637	putative transposase	**7.42**	**3.04**

**Aromatic hydrocarbon catabolism**			

BCAL0212	putative phenylacetic acid degradation NADH oxidoreductase PaaE	**2.29**	-

BCAL0213	phenylacetic acid degradation protein PaaD	**5.19**	-

BCAL0215	phenylacetic acid degradation protein PaaB	**3.31**	-

BCAL0216	phenylacetic acid degradation protein PaaA	**3.06**	-

BCAL0405	phenylacetic acid degradation protein PaaI	**2.49**	-

BCAL0406	probable enoyl-CoA hydratase PaaG	**2.54**	-

BCAL0407	beta-ketoadipyl CoA thiolase	**4.38**	-

BCAM1711	phenylacetate-coenzyme A ligase paaK	**3.44**	-

BCAM0058	3-oxoadipate CoA-transferase subunit A	-	**61.45**

BCAM0059	3-oxoadipate CoA-transferase subunit B	-	**70.43**

BCAM0060	3-carboxy-cis, cis-muconate cycloisomerase	-	**30.44**

BCAM0061	putative 3-oxoadipate enol-lactonase I	-	**37.36**

BCAM0062	4-carboxymuconolactone decarboxylase	-	**48.08**

BCAM0063	putative 4-hydroxybenzoate transporter	-	**5.79**

BCAM2568	putative beta-ketoadipyl CoA thiolase	-	**35.88**

BCAM2569	IclR family regulatory protein	-	**13.08**

**Miscellaneous genes**			

BCAL0297	putative thiamine biosynthesis oxidoreductase	**5.69**	**2.50**

BCAL1104	thiamine biosynthesis protein	**4.24**	**3.01**

BCAL1756	Putative metal dependent phosphohydrolase	-	1.97

BCAL3152	putative RNA polymerase sigma factor, ECF subfamily	**5.26**	**-8.93**

BCAL3153	putative lipoprotein	**6.89**	**-13.04**

BCAM0186	lectin	**7.89**	-

BCAS0125	conserved hypothetical protein	**31.75**	-

BCAS0126	MarR family regulatory protein/acetyltransferase	**9.18**	-

BCAS0292	conserved hypothetical protein	**3.98**	-

Both the outbreak isolates had upregulated genes involved in transport and efflux, restriction modification, and transposition as follows (Table 5). An ABC membrane transport protein (BCAS0081; 41-fold) and adjacent hypothetical protein with possible hydrolytic activity (BCAS0082; 35-fold) were highly expressed. The transport protein BCAS0081 contains both an ATP binding cassette and transmembrane components, and has homology to the *mdlB *gene of *E. coli *which is implicated in multidrug resistance [45]. Deletion of BCAS0081 in mutant K56-2ΔS0081 resulted in increased susceptibility to tetracycline and chlorhexidine, but did not alter the MIC for the other antimicrobial agents tested (Table 4). These data validate the microarray observed upregulation of BCAS0081 and demonstrate it does play a role in the antimicrobial resistance of *B. cenocepacia*.

Also highly upregulated in both strains were genes of a type I restriction modification system (BCAL0414-0420; Table 4). In J2315, the first gene in this operon is a pseudogene mutated with an insertion sequence (IS) that may prevent expression of the entire downstream gene cluster. PCR amplification and sequence analysis of this region revealed that BCC1616 and BCC1617 do not possess the same IS mutation, hence allowing overexpression of the genes as detected by the microarray analysis. Mutation of the putative DNA methylase, BCAL0417, in this cluster results in the attenuation of *B. cenocepacia *virulence in a rat lung model of chronic lung infection (mutant 16_E1 [46]). The DNA modification conferred by the gene cluster may also play a role in surviving the damage caused by hydroxyl radicals which are released when bacteria are exposed to bactericidal antibiotics [47]. Since the type I restriction modification cluster is clearly transcriptionally activated in the outbreak strains, it will be interesting to follow up if their expression has provided them with a resistance and virulence advantage compared to J2315 as an ancestral ET12 isolate.

Another response conserved between the two outbreak isolates was the upregulation of transposase encoding genes within ISBcen9 (multiple probes were on the microarray; see BCAL0044 and BCAM2637, Table 5). The IS profiles of *B. cenocepacia *isolates of the same MLST strain type have been recently shown to vary markedly and oxidative stress was linked to this IS movement [48]. The upregulation of the ISBcen9 transposase in these recent outbreak isolates may indicate that these strains are hypermutable with regard to this IS. This may allow very rapid phenotypic switches to occur within individual bacteria in the infecting population and alter their pathogenicity.

When examined individually, the two outbreak isolates demonstrated several global gene expression characteristics that had been observed in the antibiotic resistant *B. cenocepacia *J2315 derivatives. BCC1616 upregulated the PA degradation enzyme genes (Table 5), that were also constitutively upregulated in J2315-M (Table 2). These data re-enforce the hypothesis that the ability of *B. cenocepacia *to switch metabolism to alternative pathways in terms of aromatic hydrocarbon degradation plays a critical role in its spontaneous antibiotic resistance. It had also upregulated the class A beta-lactamase (BCAM2165) that was induced in J2315 and J2315-M upon meropenem exposure (Table 3). Phenotypically, BCC1616 possessed the same elevated resistance to beta-lactam antibiotics that J2315-M had developed after meropenem selection (Table 1), and its meropenem MIC could be reduced by growth in the presence glucose (data not shown). Genes found upregulated in the amikacin-resistant J2315-A derivative (BCAL3148-3152; Table 5) were also constitutively over-expressed in BCC1616, again correlated to high aminoglycoside resistance of this isolate (Table 1).

Several differentially expressed genes were specific to isolate BCC1617. Genes involved in the p-hydroxybenzoate degradation pathway were highly upregulated (BCAL0057-0063, BCAM2568, and the *IclR *family positive regulator protein BCAM2569; Table 5). Since the PA pathway genes were not significantly altered in BCC1617, it would be interesting to follow up if the p-hydroxybenzoate degradation pathway may provide an alternative metabolic shift for *B. cenocepacia *to evolve its antibiotic resistance. A specific resistance determinant upregulated in BCC1617 was an efflux pump (BCAL1674; Table 5) that is a homologue of the AmrAB [49] and of BpeAB [50] systems encoded by *B. pseudomallei*. Both the latter systems mediate resistance to aminoglycosides and macrolides in *B. pseudomallei *and it was notable that BCC1617 possessed very high levels of resistance to tobramycin, azithromycin and erythromycin (Table 1). An adjacent gene BCAL1672, encoding a transcriptional regulator and inactive in J2315 due to a frameshift mutation, does not have the frameshift in BCC1617 as revealed by sequence analysis. Hence, this could in turn allow the upregulation of the efflux pump in the outbreak strain compared to J2315.

BCC1617 also over expressed the phosphohydrolase BCAL1756 which was stably upregulated in the J2315-A amikacin exposed mutant. Other factors possibly contributing to the overall antibiotic resistance in BCC1617 compared to J2315 is the absence of a frameshift mutation in gene BCAL3259, a transport protein annotated as tetracycline resistance protein, and absence of an insertion sequence in BCAM1251, annotated as multidrug resistance transporter. Both latter genes are pseudogenes in J2315 [6] and also in BCC1616 (data not shown). Although their expression did not change in BCC1617, by just being functionally expressed they may mediate increased resistance to antibiotics.

## Conclusions

Our novel transcriptomic analysis of the response of *B. cenocepacia *to antibiotic exposure has demonstrated that it expresses a multitude of genes and different pathways to achieve high levels of resistance. *B. cenocepacia *J2315 and the clonally related strains we studied are phenotypically and transcriptomically very versatile and can adapt quickly to environmental conditions. A key finding was that once certain antibiotic resistance phenotypes in *B. cenocepacia *had been selected by a single antibiotic, the changes in the transcriptome were not transient, and were stably maintained in the spontaneous antibiotic resistant mutants. This finding suggests that mutations selected in the spontaneously resistant *B. cenocepacia *J2315 mutants are stably inherited and do not revert at a high frequency. A change in antibiotic therapy may therefore not reverse a previously acquired *B. cenocepacia *spontaneous antibiotic resistance trait. Our molecular analysis corroborates the results of antibiotic susceptibility testing that demonstrate focussing on a single antibiotic or antibiotic class for therapy has limited efficacy and that multiple antibiotic combinations [14] offer the best means to combat the spontaneous resistance of this pathogen. We have identified multiple novel molecular pathways *B. cenocepacia *utilises to resist sub-MIC levels of antibiotic. However, for many of the genes altering expression in relation to antibiotic exposure we could not prove a direct involvement resistance, with only 3 of the 8 *B. cenocepacia *antibiotic resistance gene deletion mutants demonstrating increased antimicrobial susceptibility. For spontaneous resistance to single antibiotics, *B. cenocepacia *expresses set of genes which are distinct from those altered by transient sub-MIC exposure, and which are stably altered in expression irrespective of the presence of the priming antibiotic. These findings suggest that spontaneous antibiotic resistance in *B. cenocepacia *selects multiple individual as well as pleiotropic mutations, stably altering the expression of many genetic pathways; uncovering the location of these mutations by genome re-sequencing and polymorphism analysis in future studies would shed light on why these changes are stably inherited traits in *B. cenocepacia*. Of the *B. cenocepacia *resistance mechanisms identified, those such as the beta-lactamases, phosphohydrolases, novel efflux pumps and phenylacetic acid pathways genes may be targeted to improve the efficacy of current antibiotics. We may also be able to harness the phenomenon of bacterial catabolite repression by glucose as a novel means to improve the efficacy of aerosolised antibiotics against certain *B. cenocepacia *during CF infection.

## Methods

### Bacterial strains and growth

*B. cenocepacia *strains J2315, K56-2, BCC1616 and BCC1617, *B. vietnamiensis *G4, *Escherichia coli *strains and plasmids used in this study are shown in Additional file 1, Table S2. Bacteria were grown on Iso-sensitest agar/broth (Oxoid, Basingstoke, UK), Tryptic Soya agar/broth (TSA/TSB; Oxoid, Basingstoke, UK) Luria Bertani agar/broth (LBA/LB; Sigma-Aldrich, St. Louis, MO, USA) or minimal medium (BSM [51]) as required. Strains BCC1616 and BCC1617 were from an outbreak of *B. cenocepacia *ET12 infection among CF patients that occurred in 2008 [15] and were confirmed to be the same sequence type as J2315 (ST-28 [6]) by sequence analysis of the *phaC*, *gyrB *and *trpB *genes [52]. The presence or absence of pseudogenes in BCC1616/1617 in comparison to strain J2315 was also determined using PCR (primer sequences are shown in Additional file 1, Table S3) and sequencing. *P. aeruginosa *ATCC 27853 was used as an antibiotic susceptible reference strain for antimicrobial analysis. LB was supplemented with 20 mM glucose for testing of catabolite repression and antibiotic susceptibility as described below. Bacterial stocks were maintained at -80°C as suspensions of fresh plate growth in TSB containing 8% dimethyl sulphoxide.

Microbial growth characteristics were determined using a Bioscreen Automated Microbial Growth Analyzer (Bioscreen C, Oy Growth Curves AB, Finland). Cells were grown in honeycomb microtiter plates at 37°C with 200 μl medium per well and an inoculum of 5 × 10^5 ^colony forming units (CFU) per ml. Optical density was measured every 5 min at 420-580 nm wideband after shaking for 5 seconds. Swimming motility [18] was determined in Iso-sensitest medium with 0.3% agar. Triplicate cultures were grown in each experiment and data from two biological replicates analyzed.

Spontaneous mutants with elevated antibiotic resistance were selected by plating strain J2315 onto Iso-sensitest agar containing antibiotic concentrations that were approximately double the MIC as follows: Amikacin (AMK) 300 mg/L; meropenem (MEM) 15 mg/L; and trimethoprim (TMP) in combination with sulfamethoxazole (SMX), 100 and 500 mg/L, respectively. The mutation frequency was calculated by dividing the total number spontaneous mutants by the total number of viable cells plated onto each selective agar. Single colonies were purified by plating, and five subsequent growth passages on agar containing the elevated antibiotic concentrations were performed prior to storage of the spontaneous antibiotic resistant mutant.

### Determination of miminum inhibitory concentrations

MICs of antibiotics were determined using a broth microdilution technique in 96-well microtiter plates according to the British Society for Antimicrobial Chemotherapy (BSAC) guidelines [53]. Antibiotics were obtained from Sigma (amikacin, clavulanic acid potassium salt, erythromycin, gentamycin, levofloxacin, piperacillin sodium, rifampicin, sulfamethoxazole, tetracycline, tobramycin, trimethoprim), Fluka (azithromycin, ciprofloxacin) or MP Biomedicals (chloramphenicol). Meropenem was obtained from Chemos and ceftazidime from Sandoz. Clarofan from Aventis was used as a source of cefotaxime, Primaxin IV from Merck as a source of imipenem, and Azactam from Bristol-Myers Squibb for aztreonam. The non-antibiotic cationic drugs theophylline, chlorpromazine and perchlorperazine were all obtained from Sigma. For the spontaneous J2315 mutants, the antibiotic on which they were selected was not included in the MIC assays.

Microtiter plates containing 200 μl of Iso-sensitest broth per well were inoculated with 5 × 10^5 ^CFU/ml of bacteria obtained by dilution of a fresh overnight culture. After incubation (routinely 24 h, but up to 40 h for slow growing isolates) at 37°C (with rotary shaking at 200 rpm) growth was monitored spectrophotometrically (630 nm) using a microtiter plate reader (MRX-TC Revelation, Dynex Technologies, Worthing, UK). Wells with an O.D. >0.1 were scored as positive for growth. The BSAC guidelines were followed for determining breakpoint sensitivities [54]. To evaluate additive or synergistic antibiotic effects of two compounds in combination, a checkerboard microdilution assay with 1.5-fold dilution steps was used. The mean fractional inhibitory concentraction (FIC) index was calculated according to standard procedures. Synergy was defined as an FIC index of ≤ 0.5, additivity or indifference was defined as an FIC index of >0.5 to 4 [55].

### Microarray analysis

Bacteria were cultivated for the microarray experiments in 25 ml Iso-sensitest medium held in 250 ml flasks; each was inoculated with 5 × 10^8 ^CFU and incubated at 37°C on a rotary shaker (150 rpm). Antibiotics and chlorpromazine were added at sub-inhibitory concentrations at the time of inoculation (the concentrations are given in Table 2 and Figure 1). The concentrations were chosen because they resulted in a 20% reduction of growth rate, this ensuring the bacteria were affected by the antibiotics but not in a lethal way. In the case of spontaneous mutants, experiments were performed at two different antibiotic concentrations: the same concentrations as used for the wild type (which did not alter the growth rate of the mutants significantly), and a higher concentration, again chosen to reduce the growth rate of each mutant by 20%. Growth was monitored spectrophotometrically and the bacteria harvested at an O.D. of 0.5 (approximately 5 × 10^8 ^CFU/ml). Cultures were swiftly aliquoted into microcentrifuge tubes and snap-cooled in liquid nitrogen before centrifuging at 20.000 × g at 4°C for 1 min. Pellets were immediately frozen at -80°C and RNA was extracted within one week of harvest using the RiboPure Bacteria Kit (Ambion/Applied Biosystems) including the optional DNase I treatment of the kit. LiCl precipitation (Ambion/Applied Biosystems) was used to concentrate the RNA and its quality was assessed with a Bioanalyzer using the RNA 6000 Nano kit (Agilent).

RNA was indirectly labelled with the CyScribe Post-Labelling Kit (GE Healthcare), with the first strand cDNA generated using random nonamers and incorporating amino allyl-dUTPs, which were then chemically labelled in a second step to minimise any dye bias. 10 μg of total RNA were used per labelling reaction and Spike-In controls (Agilent) were added to the labelling mix. *B. cenocepacia *genomic DNA was extracted as described [56] and labelled with the CyScribe Array CGH Genomic DNA Labeling System (GE Healthcare) at 1 μg per labelling reaction. A reference design was used for the microarray experiments with Cy3 labelled reference RNA or genomic DNA run as the control channel for all experiments. All experiments were performed with three biological replicates, and compared to three biological replicates of a J2315 control grown on Iso-sensitest medium without any antibiotics.

A custom microarray for *B. cenocepacia *J2315 was used, [10] however, the design was updated with each probes printed four times in a randomised distribution using the Agilent SurePrint 4 × 44K microarray platform (design AMADID#017397; ArrayExpress http://www.ebi.ac.uk/arrayexpress accession number A-MEXP-1613). Microarrays were hybridized according to the Two-Colour Microarray Based Gene Expression Analysis protocol (Agilent), except that it was adjusted for the use of cDNA by omitting the fragmentation step. Labelled experimental cDNA was used at 825 ng per microarray and labelled control genomic DNA was used at 200 ng per microarray. Hybridization, washing and scanning was performed as described in the Two-colour Microarray protocol and the data analysed using GeneSpring GX version 7.3.1.

8740 probes (7251 coding sequences [CDS] and 1489 intergenic [IG]) specific to J2315 were evaluated as follows: (i) unreliable features were filtered out (features had to have a p-value ≤ 0.05 in at least half of the samples to be used for analysis); (ii) a filter on 1.5-fold change in expression in the test condition compared to the control condition (wild type without antibiotics) was applied, and (iii) a one-way ANOVA was carried out on the resulting gene lists, using a Welch t-test with 5% false discovery rate (and without multiple testing correction). An initial cut-off of 2-fold change was used for global analysis of differential gene expression. To verify co-regulation of adjacently encoded genes, those within clusters, or those with known roles in antibiotic resistance, a more relaxed cut-off of 1.5-fold change was used and reported if found to be statistically significant. The microarray raw data is accessible in ArrayExpress (accession numbers E-MEXP-2708, -2738 and -2747) and the complete gene expression dataset (including the statistical confidence data) is available as a supplementary data spreadsheet (Additional file 2).

### qPCR for validation of microarray results

qPCR primers and annealing temperatures are listed in Additional file 1, Table S3. The correct size of amplification product and absence of unspecific product was confirmed by PCR on genomic DNA and subsequent agarose gel electrophoresis. RNA was extracted with the RiboPure Bacteria Kit (Ambion/Applied Biosystems) from newly generated biological replicates as follows: (i) one set mimicking the same culture conditions and concentrations of antibiotics used for microarray experiments; (ii) two sets of biological replicates where the culture conditions were modified and J2315 was grown on Iso-sensitest medium to O.D._600 _0.5 without drugs, before adding higher but still sub-inhibitory concentrations of drugs (AMK 250 mg/L, MEM 20 mg/L, TMP-SMX 50 and 250 mg/L, chlorpromazine 0.25 mM). This reduced the doubling time by 10 to 20%, and then the control and test cultures were harvested after further growth to O.D._600 _1.0. Extracted RNA was diluted to 150 ng/μl and treated for a second time with DNase (RQ1, Promega). cDNA was generated with the ImProm-II Reverse Transcription System (Promega) using 200 ng RNA per reaction. Real-time qPCR was performed as described in Drevinek et al. [10] using 250 ng cDNA per reaction; non-amplification, non-template, and no reverse transcriptase controls were included in all assays. Multiple reference genes were evaluated as controls for qPCR (data not shown) and BCAM0918, encoding RpoD, selected as the most stably expressed control gene as it showed minimal changes in expression across all conditions used in this study. Fold changes were calculated according to the delta delta C_T _method. qPCR was also used for investigating expression of selected genes in different strains (J2315, K56-2 and J2315-M; see below) under the following conditions: LB medium with and without glucose (20 mM), and with and without salicylate (10 mM). Growth, RNA extraction and qPCR was performed exactly as described above.

### Genetic manipulation

*B. vietnamiensis *G4 was used as an antibiotic susceptible complementation host for genes or gene clusters implicated in antibiotic resistance and with no close homologues in this strain. Genes were cloned into the arabinose inducible pMLBAD vector and introduced into *B. vietnamiensis *G4 by triparental mating [57]. The correct insert identity was confirmed by sequencing. Primer sequences for construction of the vectors are listed in Additional file 1, Table S3. Selection after triparental mating was performed on tryptone soya agar containing 75 mg/L trimethoprim and 400 U/ml polymyxin. MIC tests were performed on two transformants of each gene and gene cluster tested and compared to *B. vietnamensis *G4 transformed with pMLBAD. The test medium was supplemented with arabinose at 0.5% final concentration, no trimethoprim selection was applied during the MIC tests. *B. cenocepacia *K56-2 was used for mutagenesis experiments and unmarked deletions were constructed using the yeast homing endonuclease I-SceI, as described by Flannagan et al. [58]. Transformation was performed by electroporation. Primer sequences for construction of pGPI derivatives are listed in Additional file 1, Table S3. All mutations were confirmed by PCR and subsequent phenotypic testing was carried out on two independently isolated mutants.

### Bioinformatic analysis

Putative promoters and regulator binding sites were predicted by analysing the upstream region of upregulated genes with the motif discovery tool MEME [59]. The binding sites predicted by MEME were used for a search against genomes of other *Burkholderia *species using the MAST tool [60]. Signal peptides were predicted using SignalP 3.0 [61] and subcellular location of proteins was predicted with PSORT-B [62]. Homologues for genes across the genus *Burkholderia *were searched for by BLASTp [63] or the *Burkholderia *Genome Database [64].

## Competing interests

The authors declare that they have no competing interests.

## Authors' contributions

AS and AM performed all the experimental work. EM conceived the study and both EM and AS participated in the design of experiments. ET and JJP provided resources and information for the outbreak strains examined. AS, JJP and EM wrote the paper, and all authors have read and approved the final manuscript.

## Supplementary Material

Additional file 1 **Supplementary tables and figures**. A single document containing the following supplementary material: (i) Table S1: Fold changes in *B. cenocepacia *gene expression determined by qPCR; (ii) Table S2: Strains and plasmids used in this study; (iii) Table S3: PCR primers used in this study; and (iv) Figure S1. Swimming motility of *B. cenocepacia *J2315 and the outbreak isolates. Click here for file

Additional file 2 **Summary of significant changes in *B*. cenocepacia J2315 gene expression observed for all the conditions tested**. An Excel spreadsheet of all genes significantly altering their expression under the conditions examined is provided. Columns are provided for the systematic gene identifier (Gene ID) and its functional annotation in the *B. cenocepacia *genome [6], the fold gene expression change under the conditions examined (see Figure 1), and the statistical confidence p-value associated with each gene expression change. Click here for file
