# Supplementary material for: Spontaneous and evolutionary changes in the antibiotic resistance of Burkholderia cenocepacia observed by global gene expression analysis
Source: BMC Genomics. 2011 Jul 22;12:373. doi: 10.1186/1471-2164-12-373 (PMC3155924; doi:10.1186/1471-2164-12-373)
Supplement: Additional file 1 — Supplementary tables and figures. A single document containing the following supplementary material: (i) Table S1: Fold changes in B. cenocepacia gene expression determined by qPCR; (ii) Table S2: Strains and plasmids used in this study; (iii) Table S3: PCR primers used in this study; and (iv) Figure S1. Swimming motility of B. cenocepacia J2315 and the outbreak isolates. [file 1471-2164-12-373-S1.PDF]

Table S1: Fold changes in *B. cenocepacia* gene expression determined by qPCR

Target gene	Annotation	Antibiotic or adapted mutant	Microarrays	qPCR microarray conditions	qPCR modified conditions
**J2315 in the presence of antibiotics**					
BCAL1756	Metal dependent phosphohydrolase	AMK	1.8	0.8	5.3/2.1 [b]
BCAM0829a	Acetyl transferase	AMK	(1.4) [a]	0.8	2.4/2.1 [b]
BCAS0156	Beta-lactamase	MER	116	139	417/727 [c]
BCAS0130	ABC transporter protein	MER	- 2.9	- 6.7	-16.7/-16.7 [c]
BCAS0293	aidA	TMP	8.6	5.3	8.3/3.1 [d]
BCAS0167	Squalene-hopene cyclase	CPZ	4.4	5.7	5.1/4.4 [e]
BCAM0927	Multidrug efflux system transport protein	CPZ	13.7	364	784/516 [e]
BCAM2186	Macrolide-specific efflux system transport protein	CPZ	2.0	2.9	3.4/2.6 [e]
**Adapted J2315 mutants without antibiotics**					
BCAL3151	Membrane protein	J2315-A	2.8	14.9	n/d
BCAM0829a	Acetyl transferase	J2315-A	2.5	2.1	n/d
BCAL1756	Metal dependent phosphohydrolase	J2315-A	(1.8) [a]	2.7	n/d
BCAL0216	Phenylacetic acid degradation protein paaA	J2315-M	14.8	9.9	n/d
BCAL0408	Phenylacetic acid degradation protein paaZ	J2315-M	9.9	13.5	n/d
BCAM1711	Phenylacetic acid degradation protein paaK	J2315-M	12.4	6.1	n/d
BCAM1712	3-hydroxybutyryl-CoA dehydrogenase	J2315-M	no change	16.7	n/d
BCAM2552	Hydrolase	J2315-T	187	287	n/d
BCAS0293	aidA	J2315-T	- 5.9	- 4.8	n/d

Footnotes:
[a] Fold change in expression observed but result failed to pass the statistical filters applied
[b] Results obtained for two replicate cultures grown in the presence of 250 µg/ml amikacin
[c] Results obtained for two replicate cultures grown in the presence of 20 µg/ml meropenem
[d] Results obtained for two replicate cultures grown in the presence of 50/250 µg/ml trimethoprim/sulfamethoxazole
[e] Results obtained for two replicate cultures grown in the presence of 0.25 mM chlorpromazine

Table S2: Strains and plasmids used in this study

Strain or plasmid	Description	Source
***Burkholderia* sp. strains:**		
J2315	*B. cenocepacia*, clinical isolate, MLST type 28	[1]
J2315-A	AMK^r ,spontaneous mutant with elevated AMK resistance	This study
J2315-M	MEM^r,spontaneous mutant with elevated MEM resistance	This study
J2315-T	TMP^r,spontaneous mutant with elevated TMP/SMX resistance	This study
K56-2	*B. cenocepacia*, clinical isolate, MLST type 30	[1]
K65-2 ΔS0167	K56-2, ΔS0167 (sqalene-hopene cyclase)	This study
K65-2 ΔM2186-8	K56-2, ΔM2186-8 (efflux pump)	This study
K65-2 ΔM0924	K56-2, ΔM0924 (transcriptional regulator)	This study
K56-2 ΔM0925-7	K56-2, ΔM0925-7 (efflux pump)	This study
K56-2 ΔS0081	K56-2, ΔS0081 (ABC transporter)	
K65-2 ΔM0831	K56-2, ΔM0831 (ABC transporter)	This study
K56-2 ΔL1755-6	K56-2, ΔL1755-6 (phosphohydrolase)	This study
K56-2 ΔS0293-2	K56-2, ΔS0293-2 (function unknown)	This study
G4	*B. vietnamiensis*	[1]
G4-pMLBAD	G4, pMLBAD	This study
G4-S0156	G4, pLMBAD-S0156 (beta-lactamase)	This study
G4-M0829a	G4, pMLBAD-M0829a (acetyltransferase)	This study
G4-L1755+6	G4, pMLBAD-L1755+6 (phosphohydrolase)	This study
G4-M2186-8	G4, pMLBAD-M2186-8 (efflux pump)	This study
K65-2-pMLBAD	G4, pMLBAD	This study
K65-2-S0156	G4, pLMBAD-S0156 (beta-lactamase)	This study
K65-2-M0829a	G4, pMLBAD-M0829a (acetyltransferase)	This study
K65-2-L1755+6	G4, pMLBAD-L1755+6 (phosphohydrolase)	This study
K65-2-M2186-8	G4, pMLBAD-M2186-8 (efflux pump)	This study

BCC1616	*B. cenocepacia*, clinical isolate, MLST type 28	[2]
BCC1617	*B. cenocepacia*, clinical isolate, MLST type 28	[2]
***E. coli* strains:**		
E. coli OmniMAX^{TM} 2-TI^{R}	F′ {*pro*AB+ *lac*Iq *lac*ZΔM15 *Tn*10(TetR) Δ(*ccd*AB)} *mcr*A Δ(*mrr-hsd*RMS-*mcr*BC) φ80(*lac*Z)ΔM15 Δ(*lac*ZYA-*arg*F) U169 *end*A1 *rec*A1 *sup*E44 *thi*-1 *gyr*A96 *rel*A1 *ton*A *pan*D	Invitrogen
E. coli Dh5α	F^{-}, Φ80 *lac*ZΔM15 *end*A1 *rec*A1 *hsd*R17(rK^{-} mK^{+}) *sup*E44 *thi*-1Δ*gyr*A96 (Δ*lac*ZYA-*arg*F)U169 *rel*A1	[3]
E. coli SY327	*araD* Δ(*lac pro*) *argE*(Am) *recA56 nalA* λ *pir*; Rif^{r}	[4]
**plasmids:**		
pRK2013	ori_{ColE1}, RK2 derivative, Km^{r} *mob*$^{+}$ *tra*$^{+}$	[5]
pMLBAD	ori_{pBBR1}, *araC*-P_{BAD} Tp^{r} *mob*$^{+}$	[3]
pLMBAD-S0156	pMLBAD with BCAL0156, beta lactamase	This study
pMLBAD-M0829a	pMLBAD with BCAM0829a, acetyl transferase	This study
pMLBAD-L1755+6	pMLBAD with BCAL1755 and 1756, phosphohydrolase	This study
pMLBAD-M2186-8	pMLBAD with BCAM2186 to 2188, efflux pump	This study
pGPI-SceI	ori_{R6K}, ΩTp^{r} , *mob*$^{+}$, ISce-I restriction site	[6]
pDAI-SceI	ori_{pBBR1}, Tet^{r}, *mob*$^{+}$, P_{dhfr}, ISce-I homing endonuclease	[6]
pGPI-SceI-S0167	pGPISce-I with upstream flanking region of BCAS0167::downstream flanking region of BCAS0167	This study
pGPI-SceI-M0924	pGPISce-I with upstream flanking region of BCAM0924::downstream flanking region of BCAM0924	This study
pGPI-SceI-M2186-8	pGPISce-I with upstream flanking region of BCAM2186::downstream flanking region of BCAM2186	This study

References:

1. **Mahenthiralingam E, Coenye T, Chung JW, Speert DP, Govan JRW, Taylor P, Vandamme P:** Diagnostically and experimentally useful panel of strains from the *Burkholderia cepacia* complex. *J Clin Microbiology* 2000, 38(2):910-913.
2. **McIntyre K, Muller M, Ota J, Stephenson AL, Tullis E:** Epidemic of *Burkholderia cenocepacia* ET12 in the Toronto Adult Cystic Fibrosis clinic: Lessons learned. *Pediatric Pulmonology* 2009:279.
3. **Lefebre MD, Valvano MA:** Construction and evaluation of plasmid vectors optimized for constitutive and regulated gene expression in *Burkholderia cepacia* complex isolates. *Appl Environ Microbiol* 2002, 68(12):5956-5964.
4. **Miller VL, Mekalanos JJ:** A novel suicide vector and its use in construction of insertion mutations: Osmoregulation of outer membrane proteins and virulence determinants in *Vibrio cholerae* requires *toxR*. *J Bacterio* 1988, 170(6):2575-2583.
5. **Figurski DH, Helinski DR:** Replication of an origin-containing derivative of plasmid RK2 dependent on a plasmid function provided in trans. *Proc Natl Acad Sci USA* 1979, 76(4):1648-1652.
6. **Flannagan RS, Linn T, Valvano MA:** A system for the construction of targeted unmarked gene deletions in the genus *Burkholderia*. *Environ Microbio* 2008, 10(6):1652-1660.

Table S3: PCR primers used in this study

Primers used in qPCR:

Target gene	Forward and reverse primer (5' to 3')	Product size (bp)	Annealing temperature
Control gene BCAM0918	GAGATGAGCACCGATCACAC<br>CCTTCGAGGAACGACTTCAG	143	60/64 ºC
BCAL0216	CAGTCCACAGCAGTCAGTCC<br>GCTTCGTTCCATTTCAGGTC	155	64 ºC
BCAL0408	TTCACGAAGCACGAAGACAC<br>GCTGGTCGAGTACGAAGGTC	172	64 ºC
BCAL1756	CCCGACTATCCGCTGTTTC<br>CAGGTCGAGCGTCTTCTTG	282	64 ºC
BCAL3151	CGCTCGACGAATACGGTTAC<br>ATCCGACCCGAAATACGTG	191	64 ºC
BCAM0829a	CGTCGCTGACTCGCTATATG<br>CTGCTTGACGTGATGAATCC	173	64 ºC
BCAM0927	ACAACCCGAAACGACAGC<br>AGGTGAGCCGTACCCTTTG	157	64 ºC
BCAM1711	ATCTACGGGCTGTCGGAAG<br>TGATCTCCGGGTAGAAATGG	103	64 ºC
BCAM1712	GGAGAAGGGCGTGAACTATC<br>ATAGCGGTCCTCGCCATAG	115	64 ºC
BCAM2165	TCCAGTACAGCGACAATTCG<br>GCGGTATTCAGTTCGGTCTC	136	64 ºC
BCAM2186	AAGCATCCGCAGTACCTGTC<br>GCTTGACCTTCAGCGTCTTC	142	64 ºC
BCAM2552	AAGACCGGGTTGCCAGTAG<br>ACGAGAGCTGGTAAGCGTTC	113	64 ºC
BCAS0130	GAGCACCTGAAGAACGATCC<br>GGGAAGAAGTGCTTGTCGTC	263	64 ºC
BCAS0156	GCGACCTGGATCAACAAGAC<br>ACCCGAGAATCCGGTAGG	148	64 ºC
BCAS0167	GCTGCATCGACTACGTGAAG<br>TATAGGGCTGCGACTTGTCC	137	64 ºC
BCAS0293	AATATCCGAATCCCAGCAAG<br>CGTTGCCGATGAACTTGTAG	213	60 ºC

Primers for analysis of pseudogenes:

Target gene	Forward and reverse primer (5' to 3')	Mutation type	Annealing temperature
BCAL0414	TCGAGCCGGTAAAGATGAAG<br>AGGCAAGGTGATGGATGAAG	disruption by IS element	60ºC
BCAL1672	AAACGACAGCGAACTGTAAGG<br>AGCAGCGACACCAGATAAACG	frameshift	60ºC
BCAL3259	GCAGCACCGATACCCACTAC<br>GTACATGCGCAATTTCATCG	frameshift	45ºC
BCAM1251	GGGTGCTGACCTCCTACATC<br>CGACCGTGTACTGCATCATC	disruption by IS element	60ºC

Primers used for transformation-mediated complementation in *B. vietnamiensis* G4:

**Primer name**	**Forward and reverse primer (5' to 3'; restriction endonuclease sites are underlined)**	**Restriction endonuclease**	**Annealing temperature**
S0156 forward	ATAGAATTCGACAGGCCGGTCGCCAC	Eco R I	
S0156 reverse	ATATCTAGATGCTCAATCGGCTGTCC	Xba I	69ºC
M0829a forward	ATGAATTCGTCACATGACCCGGCGAC	Eco R I	
M0829a reverse	ATTCTAGAATTGCCTGGCCGACATG	Xba I	70ºC
L1755+6 forward	ATACCATGGACTCGCACTGACACCGCAAC	Nco I	
L1755+6 reverse	ATATCTAGAATCCGGAACTGAAATGAGGTGC	Xba I	67ºC
M2186-8 forward	ATACCATGGCGCCGACCGGATCGTTC	Nco I	
M2186-8 reverse	ATATCTAGAGCGCGGTGGCGTTCGCAC	Xba I	69ºC

Primers for mutagenesis of *B. cenocepacia* K56-2:

Primer name	**Forward and reverse primer (5' to 3'; restriction endonuclease sites are underlined)**	**Restriction endonuclease**	**Annealing temperature**
Pre924F	TTTTCTAGATGCATCGGGATGCGGCACGC	Xba1	71°C
Pre924R	TTTGAATTCGCTGCCGGCAAGACCCGGC	EcoR1	71°C
Post924F	TTTGAATTCGGGGCGGCCCATCGCCGGG	EcoR1	71°C
Post924R	TTTCCCGGGCCAGTTGTCCGAGCGCACGC	Sma1	71°C
Pre167F2	TTTTCTAGATCAACGCTCCATAATCCAG	Xba1	61ºC
Pre167R2	TTTGAATTCTACCTGAAGTATCACGGC	EcoR1	61ºC
Post167F	TTTGAATTCACCATCCAGCCGGAATGTTTATCTC	EcoR1	67ºC
Post167R	TTTCCCGGGTCGAGCTGCCGGACGATTTC	Sma1	67ºC
Pre2186F	TTTTCTAGACGCATGACGCAGAACATCTAC	Xba1	65ºC
Pre2186R	TTTGATATCGAGAATGCAGCGAGGATGAAG	EcoRV	65ºC
Post2188F	TTTGATATCCGGCCGTCACGCGCCGCAC	EcoRV	71ºC
Post2188R	TTTCCCGGGTCGACGTCGCGGGTATCGTCACGC	Sma1	71ºC

Primers for detection of tetracycline resistance gene of pDAISce-I

**Primer name**	**Forward and reverse primer (5' to 3')**	**Product size (bp)**	**Annealing temperature**
TetA f	CGATATCACTGATGGCGATG	526	60ºC
TetA r	GAAGGCAAGCAGGATGTAGC		

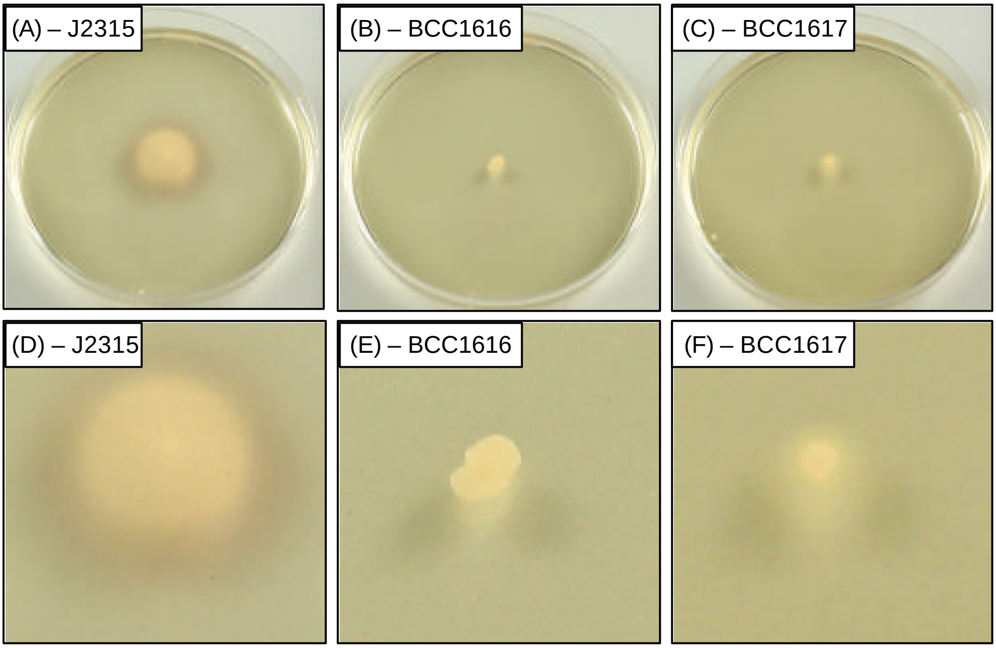

Figure S1. Swimming motility of *B. cenocepacia* J2315 and the outbreak isolates. Bacteria were stabbed into the centre of soft Isosensitest agar plates and grown for 24 hours. The zone of swimming is shown for J2315, BCC1616 and BCC1617 in panels A, B and C respectively; panels D, E, and F show a close up of the growth and spread of each respective strain.
